# A Case of Acetaminophen Toxicity in a Patient With an Unusual Alpha-1 Antitrypsin Phenotype

**DOI:** 10.7759/cureus.88224

**Published:** 2025-07-18

**Authors:** Ashwin Agrawal, Manar Chowdhury, Colin Winkie, Chickajajur Vijay

**Affiliations:** 1 Pediatric Gastroenterology, RWJBarnabas Health, Long Branch, USA; 2 Internal Medicine, West Virginia University, Morgantown, USA; 3 Pediatric Gastroenterology, Nationwide Children’s Hospital, Columbus, USA; 4 Pediatrics, West Virginia University, Morgantown, USA

**Keywords:** acetaminophen toxicity, alpha-1 antitrypsin, drug-induced liver injury (dili), intestinal failure associated liver disease, short bowel syndrome (sbs)

## Abstract

Acetaminophen is a commonly used over-the-counter analgesic and antipyretic that can be hepatotoxic if taken in excess. We present a case of acetaminophen-mediated hepatotoxicity following ingestion of a non-toxic dose of acetaminophen in a 16-year-old male with short bowel syndrome and a remote history of severe liver dysfunction with a rare alpha-1 antitrypsin phenotype Pi*EM. The patient initially presented with nonspecific symptoms of abdominal pain, nausea, vomiting, and diarrhea. N-acetylcysteine (NAC) therapy was initiated for acetaminophen toxicity. We suspect that the patient’s susceptibility to acetaminophen-induced liver injury was likely due to underlying intestinal failure-associated liver disease that occurred as a child, as well as the Pi*EM, making the liver more prone to insults. This case highlights the importance of prompt recognition and management of acetaminophen toxicity in patients with prior liver disease, even if the amount ingested is thought to be non-toxic. In addition, the case highlights that rare alpha-1 antitrypsin phenotypes should be treated with heightened caution for liver dysfunction, as there is limited literature indicating if these phenotypes are pathogenic or non-pathogenic.

## Introduction

Acetaminophen is a known offender of liver injury. While death and hepatic abnormalities typically involve doses greater than 75 mg/kg/day, risk factors, including malnutrition, inborn errors of metabolism, and interactions with other medications, may predispose certain individuals to acetaminophen-induced hepatotoxicity at lower doses [1,2]. This case demonstrates a rare incidence of hepatotoxicity following ingestion of a non-toxic dose of acetaminophen in a susceptible patient with a history of short bowel syndrome with a rare alpha-1 antitrypsin (A1AT) phenotype Pi*EM. A modified version of the case report was presented as a poster at the 2024 meeting of the North American Society of Pediatric Gastroenterology, Hepatology, and Nutrition [3].

## Case presentation

A 16-year-old male developed a cough, subjective fevers, and chills. He took ibuprofen and acetaminophen for symptomatic relief. One day later, he developed acute right upper quadrant (RUQ) abdominal pain with nausea, vomiting, and diarrhea. Pain persisted for several hours, so the patient was brought to an emergency department (ED).

The patient’s past medical history included premature birth and necrotizing enterocolitis, resulting in the resection of 50 centimeters of small bowel. He developed short bowel syndrome and was dependent on total parenteral nutrition (TPN) until the age of two. Prior to achieving enteral autonomy, he developed severe cholestasis and liver dysfunction. For a time, he was placed on a liver transplant list. His liver dysfunction gradually resolved once TPN was discontinued, and he ultimately did not require a liver transplant.

**Figure 1:**
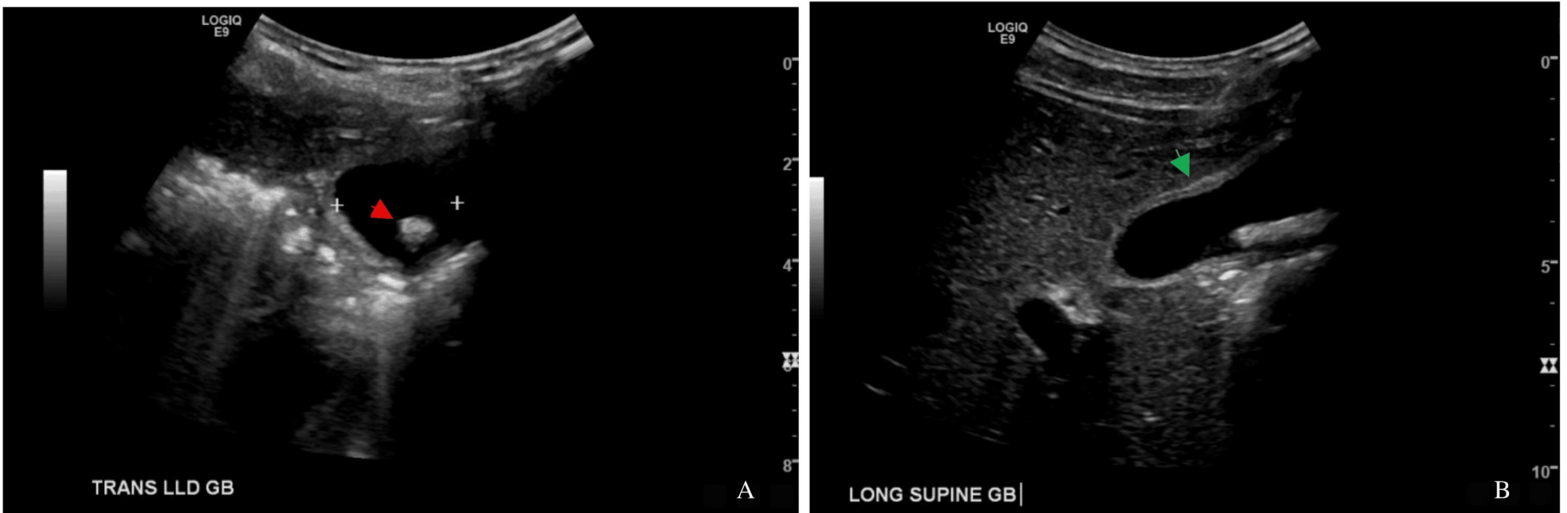
Ultrasound images of the gallbladder. A shows the gallbladder in left lateral decubitus position with a likely gallstone (red arrow); B shows the gallbladder in supine position with mild gallbladder wall thickening (green arrow).

In the ED, weight was recorded to be 50.3 kg, and he had a body mass index of 19.1 kg/m^2^. Lab work that is pertinent is shown in Table 1. A gastrointestinal viral panel was normal. Point-of-care ultrasound of the RUQ revealed gallstones and gallbladder wall thickening (Figure 1). He was admitted to the hospital and started on ceftriaxone and metronidazole for treatment of presumed cholecystitis.

**Table 1:** Initial pertinent laboratory results. The rest of the CBC was unremarkable.

Test name	Test result	Normal range
White blood cell count	20,600/mm^3^	3,899-9,800/mm^3^
Aspartate transaminase	141 U/L	14-35 U/L
Alanine transaminase	121 U/L	9-25 U/L
Direct bilirubin	0.4 mg/dL	0.1-0.4 mg/dL
Total bilirubin	2.7 mg/dL	0.1-0.8 mg/dL

The next morning, the patient was asymptomatic except for tenderness to palpation of the RUQ. Pediatric surgery evaluated the patient and believed the symptoms were from biliary colic without cholecystitis. Antibiotics were discontinued, and the patient was monitored overnight.

**Figure 2:**
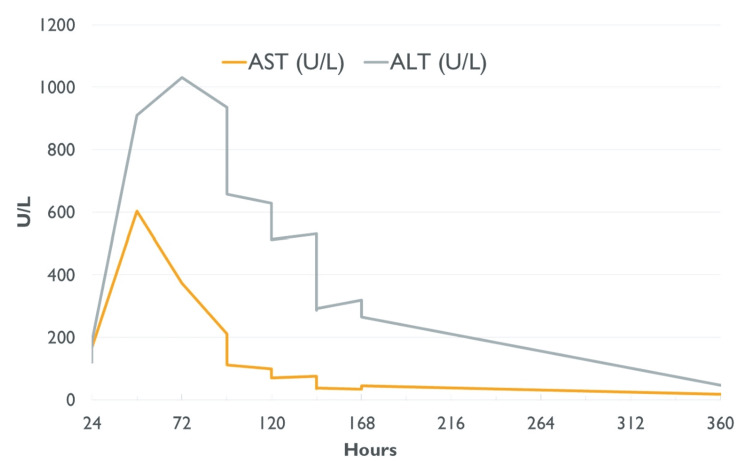
Measurements of aspartate transaminase (AST) and alanine transaminase (ALT) following ingestion of acetaminophen.

The patient remained afebrile, and abdominal pain resolved; however, aspartate transaminase (AST) and alanine transaminase (ALT) increased to 604 U/L and 911 U/L, respectively, about 46 hours after presentation (Figure 2).

Throughout the course of hospitalization, the child continued to have normal albumin with an initial value of 3.9 g/dL. His prothrombin time and international normalized ratio (INR) remained within normal limits with a max INR of 1.2. Lipase remained normal. The child's physical exam did not reveal any scleral icterus, jaundice, or signs of hepatic encephalopathy throughout hospitalization. 

Further history about acetaminophen ingestion was taken. Two days prior to the presentation, the patient reported taking four tablets of 325 mg acetaminophen at night. He took an additional six tablets at once the next morning, one day prior to the presentation. He denied any history of chronic acetaminophen use.

Acetaminophen levels were undetectable. Poison control was consulted due to concerns for acetaminophen toxicity and recommended starting N-acetylcysteine (NAC) infusions. After the first infusion, the patient developed an anaphylactoid reaction. He was treated with diphenhydramine and epinephrine, and NAC infusions were resumed at a slower rate without further issues. His ALT and AST gradually decreased (Figure 2). Table 2 shows the trends of AST, ALT, and total and direct bilirubin. Liver disease evaluation with ceruloplasmin, ferritin, autoimmune hepatitis serologies, and viral studies for hepatitis A, B, and C; Epstein-Barr virus; cytomegalovirus; respiratory viral panel; and adenovirus were unremarkable, except for A1AT phenotype PI*EM.

**Table 2:** Pertinent trends of liver enzymes and bilirubin.

Hours since ingestion	Aspartate transaminase (U/L)	Alanine transaminase (U/L)	Total bilirubin (mg/dL)	Conjugated bilirubin (mg/dL)
24	141	121	2.7	0.4
43.5	168	187	1	0.4
70	604	911	0.8	0.4
81.5	452	1021	1	0.4
87.5	374	1033	1.1	0.6
97	212	937	1.1	0.5
103	176	827	0.9	0.2
113	112	658	0.8	0.4
119	177	707	0.9	0.2
122	167	697	0.8	0.2
127	100	629	0.7	0.3
137	71	514	0.7	0.4
145	76	532	0.7	0.4
155	36	287	1.1	0.2
167	38	293	0.5	0.3
180	35	320	0.6	0.3
191	46	267	0.7	0.3
367	19	48	1.2	0.5
653.5	16	17	0.9	0.4

Due to the timing of hepatocellular injury after ingestion and negative workup for other causes of hepatocellular injury and improvement with NAC, acetaminophen was suspected as the most likely cause. In our case, our patient took a single dose of acetaminophen totaling 40 mg/kg. His total acetaminophen intake within 24 hours was 3,250 mg, or 65 mg/kg/day. Despite these doses being considered non-toxic, acetaminophen toxicity was suspected due to the timing of hepatocellular injury after ingestion (Table 3).

**Table 3:** Depicts the classic timeline of acetaminophen toxicity compared to our case presentation. NAC: N-acetylcysteine

Post-injection timeline	Classic presentation for acetaminophen toxicity	Case presentation
Stage 1 (0–24 hours)	Asymptomatic or nonspecific symptoms (nausea, vomiting, anorexia).	Nonspecific symptoms of vomiting, nausea, and anorexia.
Stage 2 (24-72 hours)	Abdominal pain. Rise in transaminases. Coagulopathy, jaundice.	Developed significant abdominal pain. Transaminases begin to rise rapidly.
Stage 3 (72-96 hours)	GI symptoms tend to improve. Transaminases peak.	GI symptoms resolved 48 hours after ingestion. Transaminases peaked around 72 hours. NAC initiated.
Stage 4 (> 96 hours)	Recovery or progression to liver failure.	Eventual normalization of transaminases.

## Discussion

This case demonstrates how acute liver injury (ALI) can be caused by a typically non-toxic dose of acetaminophen when a patient's liver is already susceptible to past insults and A1AT deficiency. 

The oral weight-based dosing for acetaminophen use in the pediatric population is 10-15 mg/kg/dose every four to six hours. The recommended maximum dosage is 75 mg/kg/day to a maximum of 4,000 mg/day [1,2]. Toxicity mostly occurs in single-dose ingestions of 250 mg/kg or over 12 g/day [4]. However, studies have shown that a dose exceeding 150 mg/kg in a pediatric patient may necessitate treatment for acetaminophen toxicity [5]. Our patient’s intake was 65 mg/kg taken over the span of 24 hours, which is within the therapeutic range.

However, in adults, therapeutic doses of acetaminophen have been associated with liver injury in patients who take acetaminophen chronically, are malnourished, chronically abuse alcohol, or have cirrhosis due to glutathione depletion or alcohol induction of CYP2E1 [6,7]. However, there's limited data on such risks in children. A systematic review of 146 cases from 1950 to 2006 in children under six years old found only six cases of hepatotoxicity at therapeutic doses (<75 mg/kg/day) [1]. Another adult study found 89 cases of liver injury with therapeutic doses of acetaminophen between 2002-2019 [6].

Our patient lacked known risk factors for acetaminophen-induced ALI identified in adult studies. However, we believe his history of infantile liver disease may have made him vulnerable to such an injury. This is because patients with intestinal failure are predisposed to developing intestinal failure-associated liver disease (IFALD). Steatosis and cholestasis are the primary contributors to IFALD, which can gradually cause end-stage liver disease [8]. A small bowel length of less than 50-100 cm can decrease secretion of fibroblast growth factor 19 (FGF19), which acts as negative feedback for bile acid synthesis. Without it, cholestatic liver injury may occur [7]. Intestinal failure often necessitates the use of parenteral nutrition (PN), which can also contribute to IFALD via steatosis [8].

Some studies suggest that liver dysfunction secondary to IFALD may persist even after stopping PN [9], which we believe occurred in this case. It is also possible that our patient has some degree of unrecognized cirrhosis. Patients with cirrhosis secondary to intestinal failure have been found to remain clinically stable for prolonged periods of time [10]. This case underscores that therapeutic acetaminophen doses should not be dismissed as a cause for ALI in patients with a history of liver disease.

Our patient also had a rare A1AT phenotype Pi*EM. A1AT deficiency results in misfolded proteins intracellular accumulation in the liver, which over time causes liver injury and, in some cases, cirrhosis and liver failure. There are more than 100 phenotypes for A1AT; however, not much is known about this phenotype. Available reports suggest that it may be non-pathogenic; however, there are scant cases of Pi*EM [11]. It is possible Pi*EM makes misfolded proteins accumulate in the liver, causing his liver to be more sensitive to toxin-induced disease.

Additionally, ALI secondary to acetaminophen takes a very characteristic pattern of symptoms and laboratory abnormalities. Our case demonstrates that paying close attention to the timeline of our patients symptoms and worsening laboratory findings helped determine that the liver injury was due to acetaminophen (Table 3). 

## Conclusions

ALI from therapeutic doses of acetaminophen in children seems to be a rare occurrence. Our case demonstrates a case of ALI with a therapeutic dose of acetaminophen due to the patient’s inherent risk factors of a history of severe liver dysfunction, IFALD, and A1AT deficiency. Our case highlights the importance of evaluating symptoms and the timeline of lab abnormalities to help determine if acetaminophen toxicity is the potential cause of elevated liver enzymes. Even if acetaminophen is taken as prescribed, drug-induced liver injury should remain of higher clinical suspicion in patients presenting with acute hepatitis with a history of liver disease. To our knowledge, this is the first case report of a patient with A1AT Pi*EM with acetaminophen toxicity. Further research is needed to determine the clinical significance of Pi*EM.

## References

[1] Heard K, Bui A, Mlynarchek SL (2014). Toxicity from repeated doses of acetaminophen in children: assessment of causality and dose in reported cases. Am J Ther.

[2] Lavonas EJ, Reynolds KM, Dart RC (2010). Therapeutic acetaminophen is not associated with liver injury in children: a systematic review. Pediatrics.

[3] Chowdhury M, Winkie C, Chickajajur V (2024). How much is too much? An unusual case of acetaminophen toxicity. Annual Meeting of North American Society of Pediatric Gastroenterology, Hepatology and Nutrition.

[4] Makin AJ, Wendon J, Williams R (1995). A 7-year experience of severe acetaminophen-induced hepatotoxicity (1987-1993). Gastroenterology.

[5] Chiew AL, Gluud C, Brok J, Buckley NA (2018). Interventions for paracetamol (acetaminophen) overdose. Cochrane Database Syst Rev.

[6] Louvet A, Ntandja Wandji LC, Lemaître E (2021). Acute liver injury with therapeutic doses of acetaminophen: a prospective study. Hepatology.

[7] Verbeeck RK (2008). Pharmacokinetics and dosage adjustment in patients with hepatic dysfunction. Eur J Clin Pharmacol.

[8] Wang J, Micic D (2021). Hepatobiliary manifestations of short bowel syndrome and intestinal failure-associated liver disease. Clin Liver Dis (Hoboken).

[9] Yang CF, Lee M, Valim C (2009). Persistent alanine aminotransferase elevations in children with parenteral nutrition-associated liver disease. J Pediatr Surg.

[10] Fullerton BS, Sparks EA, Hall AM, Duggan C, Jaksic T, Modi BP (2016). Enteral autonomy, cirrhosis, and long term transplant-free survival in pediatric intestinal failure patients. J Pediatr Surg.

[11] Akbas N, Gonzalez G, Buffone GJ, Grenache DG, Devaraj S (2016). A library of rare α1-antitrypsin (AAT) variant phenotypes to aid in the diagnosis of AAT deficiency. Am J Clin Pathol.

